# Impact of age at diagnosis on clinicopathological outcomes of oral squamous cell carcinoma patients

**DOI:** 10.12669/pjms.343.14086

**Published:** 2018

**Authors:** Nosheen Mahmood, Muhammad Hanif, Akhtar Ahmed, Qamar Jamal, Saqib, Adnan Khan

**Affiliations:** 1 Dr. Nosheen Mahmood, MBBS, M.Phil. Ziauddin Medical University, Karachi, Pakistan; 2 Dr. Muhammad Hanif, PhD. Karachi Institute of Radiotherapy and Nuclear Medicine, Karachi, Pakistan; 3 Dr. Akhtar Ahmed, FCPS. Karachi Institute of Radiotherapy and Nuclear Medicine, Karachi, Pakistan; 4 Dr. Qamar Jamal, PhD. Ziauddin Medical University, Karachi, Pakistan; 5 Mr. Saqib, M.Sc. Karachi Institute of Radiotherapy and Nuclear Medicine, Karachi, Pakistan; 6 Mr. Adnan Khan, M.Sc. Karachi Institute of Radiotherapy and Nuclear Medicine, Karachi, Pakistan

**Keywords:** Clinicopathological data, Oral cancer, Squamous cell carcinoma

## Abstract

**Objective:**

A recent trend in diagnosis of oral cancer in young age is observed, however its impact on various clinicopathological parameters needs to be explored. The aim of the current study was to compare and analyze impact of age at diagnosis with clinicopathological parameters of oral squamous cell carcinoma patients.

**Methods:**

In this cross sectional study conducted at Department of Oncology Ziauddin Hospital Karachi, we included histologically confirmed cases of oral squamous cell carcinoma. The patients were categorized as young age group (40yrs and younger) and old age group (41 yrs and above). A total of 115 patients diagnosed between 2013 and 2016 were enrolled in the study. The variables considered were age at diagnosis, sex, site of lesion, positive family history, tumor grade, stage, uric acid level and survival.

**Results:**

A statistically significant difference was observed between two age groups in overall survival, uric acid level and positive family history of cancer. No significant difference was observed in tumor location, grade and stage.

**Conclusion:**

Majority of oral cancer patients present at an advanced stage irrespective of age at diagnosis but young age has an overall improved survival. Moreover, a positive family history of cancer in young age group mandates further exploration of possible role of genetic polymorphisms which might be responsible for early onset of the disease.

## INTRODUCTION

Oral cancer is the 6^th^ commonest cancer worldwide.^1^ According to recent estimates of American Cancer Society, about 49,670 subjects are expected to develop Oral cavity or oropharyngeal cancer and 9,700 may die of it by the end of 2017. Men are two times more likely to suffer from these cancers; however, there is no difference in prevalence between blacks and whites.^2^ A retrospective study in which data collected for head and neck cancers for the period of 1998 to 2009, in London, have shown an increase in age standardized incidence rates from 17% to 40% among males and 7% to 87% among females. The study further reported cancer of the oral cavity as the second commonest site after larynx, in both the sexes. It is alarming to see that every fifth patient was from a non-white group and a higher incidence of oral cancer is noted in Indian and Pakistani population.^3^

In Pakistan oral cancer ranked as the second in incidence, prevalence and mortality among other ailments. IARC Globocan data for 2012 showed an incidence of 12761(8.6%), prevalence of 30647 (8.9%), and mortality of 7266 (7.2%). It is the commonest cancer in Pakistani males and second after breast cancer in females.^4^

Excessive consumption of Betel quid, Areca nut and chewable tobacco products is the main reason of a higher incidence and prevalence in South Asia. Oral cancer incidence increases with age and a vast majority of cases reported to be diagnosed between 50 to 60 years. However over last several years a rising trend in young age group is observed in many epidemiological studies.^5^ Byers et al. drew attention towards this rise in 1975 and later reports also confirmed these findings.^6^ Literature from Pakistan shows majority of cases are diagnosed between 41-50 years but a steady rise in incidence is observed in patients younger than 40.^7^

There is a need to consider the younger patients as a different cohort who might be differing in terms of risk factors, tumor characteristics and prognosis. Several features of early onset of oral cancer present unique opportunities for genetic and clinical research because the majority of patients diagnosed at younger ages tend to have advanced tumors but still present a better prognosis. Further exposure to most of the risk factors is same but it takes a shorter time for development of cancer in young patients and they are equally advance in terms of nodal and distant metastasis. This may be due to a complex interplay between environmental factors and genetic mutations in them.

Since early onset Oral cancer is enriched for complex interplay between genetic and environmental risk factors, future research provides opportunity to help improve our understanding of the etiology and hence open new doors for screening targeted population and provide individualized targeted therapy.

## METHODS

A formal ethical approval was obtained from local committee at Ziauddin University. A total of 115 consecutive OSCCA patients included who were referred to Department of Oncology Ziauddin Hospital Karachi, Pakistan. All cases registered between January 2013 and September 2016 was studied prospectively. Patients were included in the study after taking written informed consent. Eligible patients had histologically confirmed squamous cell carcinoma. A structured questionnaire was designed to record information on consumption of betel quid, areca nut, niswar, alcohol and smoking history. Any subjects aged 40 years or less was defined as young and old if above 40. Pathologic and clinical records of the patients were reviewed. Information including age, sex, past medical history, duration of the disease, habits, presence or absence of recurrence, tumor size, number and size of involved nodes were all registered in a structured questionnaire after conducting interview and physical examination. The primary endpoints were the impact of age at diagnosis on clinical stage, grade, and overall survival at the end of study.

Tumor staging was done according to AJCC guidelines 7^th^ edition for Oral cancers. The WHO grading system was applied to classify tumors into well, moderately or poorly differentiated.

### Statistical Analysis

Variables described as mean ± standard deviation. All data analysis was done using statistical package for social sciences, version 21 SPSS. The comparison of baseline characteristics between the CKD and control groups was done using the Chi Square (X^2^) test to analyze the qualitative variables in various groups. Quantitative variables were analyzed by t test. Multiple comparisons were made using post hoc Tukey Krammer test. Correlation between continuous variables was made by Pearson’s correlation. P-value of <0.05 was considered as significant.

## RESULTS

A total of 115 patients were analyzed including 82 (71.3%) males and 33(28.7%) females. Mean age at presentation was 45.6± 12.3 years. Majority of patients were Urdu speaking 79 (64.3%) followed by Sindhi 16 (13.9%), Balochi 7 (6.8%), Punjabi 6 (5.2%), Pashtoons 3 (2.6%) and others 4 (3.5%).

Upon grouping with respect to age, there were 40 subjects who were less than 40 years old while 75 were above 40 years of age (Table-I). Patient and tumor charectaristics in the two age groups are summarized in Table-I. Patients with oral cancer were more likely to be male (71% vs. 29%, p = 0.003). The male/ female ratio in the young and old group was 4.7/1 and 1.88/1 respectively. The mean age was 32.5 in young and 52.63 years in the older group. The youngest male and female were 20 and 27 years old respectively.

**Table-I:** Distribution of age at diagnosis in relation to patient and tumor characteristics.

		Age ≤ 40 (n=40)	Age >40 (n=75)	Total (n=115)	p-value
Gender	Male	33	49	82	0.053
	Female	7	26	33	
Site	ICD 10-00	1	4	5	0.720
	ICD 10-01	0	1	1	
	ICD 10-02	7	12	19	
	ICD 10-05	1	5	6	
	ICD 10-06	31	53	84	
Family history of Oral Cancer	No	32	73	105	0.002**
	Yes	8	2	10	
Tumor size	T1	2	2	4	0.368
	T2	22	34	56	
	T3	8	15	23	
	T4	8	24	32	
Nodal metastasis	N0	5	12	17	0.815
	N1	9	14	13	
	N2	26	49	75	
Metastasis	No metastasis	38	70	108	0.722
	Distant metastasis	2	5	7	
Tumor Grade	Well differentiated	5	12	17	0.038*
	Moderately differentiated	24	56	80	
	Poorly differentiated	11	7	18	
Tumor Stage	I	1	0	1	0.221
	II	1	8	9	
	III	10	15	25	
	IV	28	52	80	

* p< 0.05,

** p <0.01,

ICD 10-00;Lip cancer, ICD 10-01; Cancer Base of Tongue, ICD 10-02;

Other non specified parts of tongue, ICD 10-03; Gum, ICD 10-04 ; Floor of mouth, ICD 10-05;

Palate, ICD 10-06; un specified parts of mouth.

Upon sub grouping there were 16 (13.9%) subjects less than 30, 24 (20.9%) between 31 -40, 37 (32.2%), between 41-50, 25 (21.7%), between 50 to 60 and 12 (10.4%) above 61.

There were 80 (69.6%) patients in stage III, 25 (21.7%) in Stage IV, 9 (7.8%) in stage II and only 1 (0.9%) in stage 1. Tumor grading showed 80 subjects (69.6%) presented had moderately differentiated cancer, 17 (14.8%) well differentiated and 18 (15.7%) poorly differentiated tumor. Upon comparing the tumor grade in the two groups a statistically significant difference was observed with a *X*^2^ =6.523 and p value of 0.038. Well differentiated tumors had a comparable distribution between the two groups with 12.5% of all tumor in younger and 16% in older group having well differentiated histology. However moderately and poorly differentiated cancer distribution was different in the two groups. In older group 74.7% were moderately differentiated tumors and 9.3% poorly differentiated whereas younger group had 60% and 27.5% poorly differentiated. Serum uric acid level before start of any treatment was available for 32 subjects ≤ 40 years age and 62 subjects>40 years. Mean serum uric acid for the two groups was 2.53 ±0.54 and 3.98 ±0.837 respectively which was statistically significant at a p-value of< 0.01.

**Fig.1:**
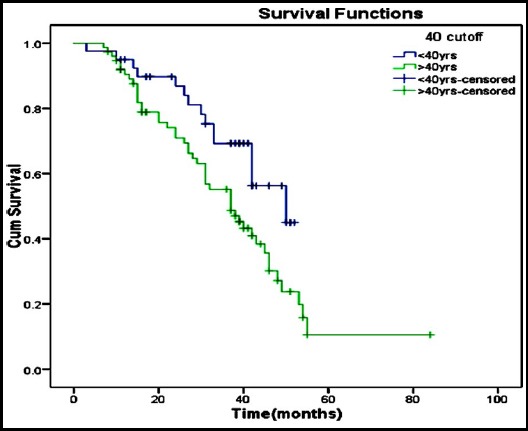
Overall survival is 46.08% with a survival of 62.5% in less than 40 years and 37.3% in above 40 years. A log rank test run to determine difference in survival distribution between <40 years old and = 40 years old was statistically significantly different, X2=4.827, p=0.027.

Overall survival rate of patients is shown in Fig.1.

## DISCUSSION

Age is considered as an important prognostic factor in cancers. In the present study majority of our patients were in 41- 50 years age bracket with a mean age of 45.6 years. Previous report from Mirza et al. exhibited that most of patients suffering from oral cancer were 51-56 years old.^8^ Similarly, Burguri et al. observed patients of oral cancer diagnosed between 50 to 69 years.^9^ Surprisingly a changing trend with most patients presenting between 41-50 years was observed by Akram et al.^7^ Alamgir et al. also observed most of their patients lied in 41-50 years age group.^10^ There is a clear shift towards diagnosis at younger age although most patients were between 40 to 50 years but a large number of patients are being diagnosed at a younger age. Sarwar et al. reviewed data of IARC Globocan 2012 for oral cavity and lip cancer and report that maximum number of patients diagnosed were in 15-39 years age bracket and these findings were present in both males and females.^11^ Exposure to smokeless tobacco, betel quid and areca nut at a younger age due to easy access and affordable price are important contributing factors.

**Table-II:** Distribution of tumor grade with different stages tumor stage.

		1	2	3	4	Total
Tumor Grade	Well Differentiated	0	2	4	11	17
	Moderately Differentiated	1	5	19	55	80
	Poorly Differentiated	0	2	2	14	18
	Total	1	9	25	80	115

In our study, 34% patients were below 40 years. Previously, Samreen at al. observed that 21.8% of their patients were less than 40 years old.^12^ Data from IARC Globocan 2012 shows that 22% males whereas 16% of females are less than 40 years old at the time of diagnosis.^11^ The current study exhibit that buccal mucosa was the most commonly site involved which is in agreement with previous studies reported from this region.^7^,^9^,^11^

Features like tumor size, nodal and distant metastasis did not show any significant difference between young and old patients. Kuriakose et al. observed an early spread to lymph nodes in their younger patients while older patients had a late lymph node invasion.^13^ Majority of our patients were in Stage III and IV. Alamgir et al. found most of their patients in Stage II and IV.^10^ In contrast Samreen et al. observed majority of patients presented in Stage II.^12^ In this cohort study there was no significant correlation of tumor grade with stage as despite an advanced stage most of the tumors were moderately differentiated. But upon comparing Grades between young and old groups we observed younger patients to have a greater proportion of poorly differentiated histology compared to older group. Contrasting findings were observed by Frare et al who found no difference in tumor grade between young and old patients, but the younger group had higher rate of nodal metastasis and tumor recurrence.^14^ Rich lymph vascular supply of oral cavity allows early tumor spread to the lymph nodes even if the tumor is well to moderately differentiate.

We observed a significant association of family history of oral cancer in our younger age group. Brown et al observed a 2.5 fold excess risk in those subjects with a family history of oral cancer in a first degree relative. A positive family history was recorded in 18.64% of their cases and 8.19% of controls. (p=0.002, OR: 2.568, 95% CI: 1.411-4.675).^15^ Regarding association of family history with age at diagnosis of cancer some studies have found higher association in young patients,^16^,^17^ while few have reported an association with older age group.^18^ In contrast to our observation Brown et al found a stronger association between the risk of oral cavity cancer and family history of head and neck cancer in subjects aged above 45.^15^

We observed an improved overall survival in younger patients. Literature search shows that earlier studies have reported poor prognosis and aggressive behavior in younger patients however, recent work shows a better survival in young patient which they have linked to a higher incidence of Human Papilloma Virus infection over recent years.^19^ It has been observed in IARC Globocan report 2012 that maximum number of new cases as well as maximum mortality was seen in less than 40 years old patients for males but for females although maximum diagnosis were made in less than 40 years but highest mortality was observed in 55-64 year old age group patients.^11^ The apparent increased mortality in older age may be the result of post-operative complications, poor tolerance for chemotherapy and co existing chronic diseases.

We observed a lower serum uric acid level in young OSCCA patients as compared to older age group. We did not find any study comparing uric acid level between young and older patients of OSCCA. Uric acid, an end product of metabolism has been proposed as an antioxidant. Considering this a lower level of uric acid might promote carcinogenesis by not being able to flush away the free radicals. A lower uric acid level in the patients as compared to controls was observed in a tertiary care hospital in Nigeria^20^, these findings were duplicated by Ara et al. who found a significantly low level of uric acid in cases as compared to controls.^21^ To explore the possible role in precancerous lesion Narang et al. investigated serum uric acid level in oral sub mucous fibrosis and did not detect any significant difference.^22^ A decreased level of Salivary uric acid was observed by Hanspal Sing et al. is OSCCA patients compared to controls and among patients a progressive decrease was seen in well to moderately and poorly differentiated tumors.^23^

## CONCLUSION

The observed differences in patient’s characteristics and clinical outcomes in young age group include a better survival, lower serum uric acid level and a positive family history of cancers. In recent years considerable attention has been paid on association between genes with the complex disease. Various experimental approaches for instance, genetic linkage studies, expression profiling, and pan genomic association studies have revealed the studies successful in recognizing high risk genes. The heterogenecity of the disease makes it difficult to probe out a single set of genes involved in the pathogenesis of the disease. However, based on the current observations young age should be considered as a distinct clinical entity mandating the need to explore underlying biology by identifying unique molecular characteristics.

### Authors’ Contributions

**NM and MH** conceived, designed and did statistical analysis & write up with editing of manuscript.

**AK and S** did data collection and bench working.

**AA and QJ** reviewed work and final approval of manuscript.
